# Extraction of Bioactive Compounds from *C. vulgaris* Biomass Using Deep Eutectic Solvents

**DOI:** 10.3390/molecules28010415

**Published:** 2023-01-03

**Authors:** Maria Myrto Dardavila, Sofia Pappou, Maria G. Savvidou, Vasiliki Louli, Petros Katapodis, Haralambos Stamatis, Kostis Magoulas, Epaminondas Voutsas

**Affiliations:** 1 Laboratory of Thermodynamics and Transport Phenomena, Zografou Campus, School of Chemical Engineering, National Technical University of Athens, 9 Iroon Polytechniou Str., 15780 Athens, Greece; 2 Department of Marine Sciences, University of Aegean, University Hill, Lesvos Island, 81100 Mytilene, Greece; 3 Laboratory of Biotechnology, Department of Biological Applications and Technologies, University of Ioannina, 45110 Ioannina, Greece

**Keywords:** DES, *C. vulgaris*, carotenoid content, phenolic content, antioxidant activity, RSM, analysis of variance

## Abstract

*C. vulgaris* microalgae biomass was employed for the extraction of valuable bioactive compounds with deep eutectic-based solvents (DESs). Particularly, the Choline Chloride (ChCl) based DESs, ChCl:1,2 butanediol (1:4), ChCl:ethylene glycol (1:2), and ChCl:glycerol (1:2) mixed with water at 70/30 *w*/*w* ratio were used for that purpose. The extracts’ total carotenoid (TCC) and phenolic contents (TPC), as well as their antioxidant activity (IC50), were determined within the process of identification of the most efficient solvent. This screening procedure revealed ChCl:1,2 butanediol (1:4)/H_2_O 70/30 *w*/*w* as the most compelling solvent; thus, it was employed thereafter for the extraction process optimization. Three extraction parameters, i.e., solvent-to-biomass ratio, temperature, and time were studied regarding their impact on the extract’s TCC, TPC, and IC50. For the experimental design and process optimization, the statistical tool Response Surface Methodology was used. The resulting models’ predictive capacity was confirmed experimentally by carrying out two additional extractions under conditions different from the experimental design.

## 1. Introduction

Phenolic and carotenoid compounds possess important pharmacological activities such as antioxidant, antibacterial, and anti-inflammatory [1,2,3]. The recovery of high-value biomolecules from several natural sources is largely realized with the conventional extraction process using traditional organic solvents [4]. Nevertheless, these solvents are often related to low yield efficiency and increased energy consumption, as well as toxicity, volatility, flammability, non-biodegradability, and non-renewability [5,6]. Given these drawbacks and considering the principles of Green Chemistry, new alternative solvents have been introduced for the extraction of essential biocompounds, such as switchable hydrophilicity solvents, SHS [7]; switchable ionic liquids, S-IL [8]; and deep eutectic solvents, DESs [8,9]. 

DESs, since their initial introduction by Abbott et al., 2003 [10], have dynamically emerged as means of surpassing the above-referred limitations. They can be produced by naturally occurring, biodegradable, and low-cost components via simple synthesis routes [6]. Usually, DESs consist of two components, a hydrogen bond acceptor (HBA) and a hydrogen bond donor (HBD). Choline chloride (ChCl) is the most widely studied HBA, while different polyalcohols, organic acids, sugars, and amino acids have been employed as HBDs [4,6,11,12]. The possible combinations of HBAs and HBDs are almost unlimited, giving the ability to design task-specific DESs [6,13,14]. Some of their most important characteristics are low volatility at high temperatures, selectivity, strong dissolving ability, adjustable polarity, biocompatibility, pharmaceutical acceptance, and high viscosity. These characteristics are severely influenced by the type and molar ratio of the HBAs and HBDs [13,15,16]. DESs with polyols, as HBDs in particular, exhibit lower freezing points and can even exist in a liquid state below room temperature [17]. 

DESs have been used in many scientific fields ranging from chemical synthesis to bio-catalysis and nanomaterials fabrication, as greener, eco-friendlier, and more efficient alternatives to traditional solvents [18,19,20,21,22,23,24]. The exploitation of DESs for the extraction of various non-polar and polar bioactive compounds from different natural sources has also shown an admirable trend [9,11,25,26,27,28]. It has been reported that DESs can provide higher extraction yields and stabilization capacity of the targeted biomolecules in comparison to conventional solvents [14,29,30]. 

However, high viscosity is one of the most important drawbacks in the exploitation of DESs as extractants at an industrial scale. To reduce the viscosity and enhance the mass transport phenomena of the biomolecules from the solid to the liquid, water, an abundant natural substance, is frequently added in various ratios, also influencing the polarity of the DESs and affecting the dissolution of the compounds of interest [12,31,32]. 

The extraction efficiency of the bioactive compounds is also dependent on several extraction parameters. Some of the most influential are the biomass-to-solvent ratio, the mode of agitation, the time and temperature of the extraction, the types and ratio of HBA and HBD, the viscosity of the DES [12,26,33], etc. Hence, the investigation of the most efficient extraction parameters for a given DES and natural source combination is important. 

*Chlorella vulgaris* is a microalgae strain which is consisted of 4% phenolic compounds, 2% carotenoids, 16% lipids, 10% carbohydrates, and other valuable components [34]. *Chlorella* species are verified to have one of the highest percentages of phenolic compounds and carotenoids compared to other microalgae strains [35]. The recovery of compounds with antioxidant activity, such as polyphenols and carotenoids, from *C. vulgaris* using conventional solvents is well documented [36,37,38,39]. On the contrary, the use of DESs for the same purpose has hardly been addressed. According to Mahmood et al., 2019 [40], polyol-based DESs have been found to outperform conventional solvents in terms of polyphenolic extraction efficiency, the antioxidant activity of the extracts, and the selectivity of target antioxidants from *C. vulgaris*.

In this study, *C. vulgaris* biomass extractions were conducted using deep eutectic-based solvents. The DESs ChCl:1,2 butanediol (1:4), ChCl:ethylene glycol (1:2), and ChCl:glycerol (1:2) were synthesized, and water was added to a 70/30 *w*/*w* ratio. The resulting mixtures were employed for the extractions of *C. vulgaris* under given conditions. Subsequently, the extracts’ total carotenoid and phenolic contents, as well as their antioxidant activity, were determined. The screening procedure of the DES/water mixtures resulted in the determination of the most efficient one, namely ChCl:1,2 butanediol (1:4)/H_2_O 70/30 *w*/*w*, which was employed thereafter for optimizing the extraction process. For that purpose, the influence of three important extraction parameters, namely biomass-to-solvent ratio, temperature, and time was studied regarding their impact on the recovery of carotenoids and phenolics and on the extracts’ antioxidant activity. An experimental design was implemented, and the Response Surface Methodology was employed for the process optimization. The influence of the independent parameters on each dependent one was determined through Analysis of Variance, and the resulting models were evaluated and confirmed experimentally by carrying out two additional extractions under conditions different from the experimental design. 

## 2. Results and Discussion

### 2.1. Physical Properties of DESs

The resulting viscosities and densities of the DESs and their mixtures with water (70/30 *w*/*w*) at 60 °C are included in Table 1. The reported values are the means of three measurements. The DES1/w, DES2/w, and DES3/w abbreviations correspond to the ChCl:1,2 butanediol (1:4)/water, ChCl:glycerol (1:2)/water, and ChCl:ethylene glycol (1:2)/water, respectively. It is observed that by far the most viscus DES was the ChCl:glycerol (1:2) (DES2), ChCl:1,2 butanediol (1:4) (DES1) follows, and ChCl: Ethylene glycol (1:2) (DES3) shows the lowest viscosity. In the case of polyol-based DESs, the hydrogen bonds that are formed between the HBAs and HBDs are proportional to the hydroxyl groups present in the molecules of the HBDs. The presence of more hydroxyl groups increases the intermolecular forces resulting in higher η values [15]. Indeed, the glycerol molecule (HOCH_2_CHOHCH_2_OH) has one more hydroxyl group than the diols 1,2 butanediol (HOCH_2_CHOHCH_2_CH_3_) and ethylene glycol (HOCH_2_CH_2_OH), justifying the higher η value of DES2. Moreover, the viscosity of DESs is also affected by the molecular structure (molecular weight and size) of the HBD [41]. Between the diols serving as the HBDs of DES1 and DES3, 1,2 butanediol is a larger molecule than ethylene glycol; hence, DES1 is more viscus than DES3.

As expected, water addition to the DESs led to a large reduction of their viscosities, which is due to the weakening of the hydrogen bonding between their constituents [42]. The viscosities of the DES/water mixtures followed the order that the pure DESs exhibited as well, i.e., DES2/w > DES1/w > DES3/w. The values of the viscosities of the three DES/water mixtures indicate that these can be used for industrial applications as solvents [43]. 

The measured densities of the pure DESs and their water mixtures employed in the present study are also shown in Table 1. It is observed that the density values of the pure DESs diminished according to the order: DES2 > DES1 > DES3, following the same order as for the viscosity. The DESs densities depend on the hydrogen bonds developed between the HBA and the HBD. In particular, when a larger number of hydrogen bonds are formed, the available free space in the DESs is reduced, resulting in increased density [44,45]. Therefore, the higher density value measured for DES2 is attributed to the surplus of hydroxyl functional groups found in the molecule of glycerol as compared to 1,2 butanediol and ethylene glycol. Moreover, DESs’ density is affected by the length of the alkyl chain of the HBD molecule. According to the literature, a longer alkyl chain results in lower densities [46,47]. This conclusion is confirmed by our research, too, since DES1 has a longer alkyl chain than DES3. 

DES2/w and DES3/w demonstrated lower densities in comparison to pure DES2 and DES3 due to the weakening of the hydrogen bond network caused by water addition [46]. However, the level of the reduction is low; thus, it can be claimed that the specific physical property of these two DESs is not significantly affected by water, at least for the given water ratio and temperature. Florindo et al. [46] came to the same conclusion for five different choline chloride-based DESs. 

The density of DES1/w was found to be marginally higher (0.0025 g cm^−3^) than that of DES1. This implies a positive excess molar volume (V^E^) upon mixing of ChCl:1,2 butanediol (1:4) with water at a 70/30 *w*/*w* ratio and 60 °C, pointing to volume compression, hence density increase. Such a phenomenon hints at stronger intramolecular interactions (i.e., among DES1 molecules or among water molecules) than interspecies interactions (i.e., between water and DES1 molecules) for the specific composition of the DES/water mixture and temperature [48]. Further investigation of the ChCl:1,2 butanediol (1:4)/H_2_O mixtures within the whole compositional range and also within a vast temperature range should be performed to obtain a clear view of the key physical property of density for this mixture. Such a study exceeds the scope of the present work and is planned for the near future.

### 2.2. Solvent Screening

The three DES-based solvents, i.e., DES1/w, DES2/w, and DES3/w, were compared for their capacity to extract bioactive compounds from *C. vulgaris*, while the EtOH/w mixture served as the control solvent. The measured TCC, TPC, and IC50 values of the obtained extracts are included in Table 2. 

According to the results of Table 2, the DES1/w solvent outperformed the two other DES-based ones in extracting carotenoid compounds from *C. vulgaris* biomass. In fact, the TCC value measured for the DES1/w extract was 93.7% and 91.5% greater than the corresponding values measured for the DES2/w and DES3/w extracts, respectively. The control solvent is the best among the four tested for carotenoid extraction. This finding is in accordance with the literature since ethanol/water mixtures are known to be particularly efficient solvents in extracting carotenoids from micro and macro algae [37,49,50]. 

The superiority of the DES1/w solvent regarding the extraction of phenolics from *C. vulgaris* biomass can be observed in Table 2. The TPC value that was determined for the DES1/w extracts was almost double the TPC values found at the DES2/w and DES3/w *C. vulgaris* extracts. In comparison to the extract obtained from the control solvent, the DES1/w delivered a total phenolics content greater by about 10%. It is also noticed that all the DES-based solvents used in the present study performed significantly better in extracting phenolic compounds than carotenoids. Several DES/water mixtures have been acknowledged for their efficiency in extracting phenolic compounds from various natural sources [5,16,35]. According to the generally accepted concept known as “like–dissolve–like”, it is suggested that the polar DES-based solvents perform better in extracting polar species, such as phenolics, than non-polar, such as carotenoids [35]. Moreover, the high extractability of phenolic compounds from DESs and their water mixtures has been attributed to the H-bonding interactions that can be formed between the phenolic molecules and those of the DESs [42]. 

Mahmood et al., 2019 [38] used different polyol-based DESs for the extraction of polyphenols from *C. vulgaris*, among which the ChCl:glycerol (1:2) and ChCl:ethylene glycol (1:2). The TPC value reported by the same authors for the ChCl:glycerol (1:2), extract (5.27 mg_GAE_ g^–1^_DW_) is comparable to the TPC measured at the ChCl:glycerol (1:2)/H_2_O 70/30 (DES2/w) extract of the present study. Moreover, the ChCl:ethylene glycol (1:2)/H_2_O 70/30 (DES3/w) extract of the present work exhibited approximately 5 times higher total phenolics content in comparison to the ChCl:ethylene glycol (1:2) extract reported by Mahmood et al., 2019 [40]. However, it should be mentioned that a direct comparison of results given by different studies of microalgae biomass extractions is rather difficult. The biochemical composition and other characteristics of microalgae biomass can differ significantly due to the type of the cultivated strain, the growth conditions, the growth phase, and the composition of the cultivating medium. Additionally, other parameters, such as the biomass drying method used, its treatment prior to the extraction, and of course, the extraction conditions and the solvent used can have a significant impact on the obtained extracts’ composition and antioxidant activity [50,51,52]. However, it can be claimed that the addition of water in ChCl:ethylene glycol (1:2) positively affected the extracts’ total phenolic content. This can be attributed to the selective extraction of water-soluble phenolic compounds present in *C. vulgaris* biomass [53]. 

The extract obtained from the conventional solvent EtOH/w exhibited the smallest IC50 value (Table 2), hence the highest antioxidant activity. The DES-based solvents follow the order DES1/w > DES2/w > DES3/w as per their antioxidant activity. Carotenoids and phenolics are potent antioxidants, and the contribution of both these bioactive compounds in the measured antioxidant activity of microalgae extracts is significant [50,52]. Considering that the DES1/w delivered an extract with considerably higher TCC and TPC values in comparison to the other two DES-based solvents, its superior antioxidant activity can be justified. Despite the higher phenolics yield exhibited by the DES1/w extract in comparison to EtOH/w one, its lower carotenoid content seems to have an impact on the measured IC50 value.

According to the solvent screening results analyzed above, the DES1/w was proven to be the most convenient for the purpose of our study between the three DES-based solvents that were tested. As mentioned, it led to the *C. vulgaris* extract with the highest content of carotenoid and phenolic compounds and the highest antioxidant activity. Consequently, the ChCl:1,2 butanediol (1:4)/H_2_O 70/30 *w*/*w* solvent was further exploited for the optimization of the *C. vulgaris* microalgae extraction process.

### 2.3. Experimental Design Results 

The experimental results of *C. vulgaris* biomass extractions performed using as a solvent the mixture ChCl:1,2 butanediol (1:4)/H_2_O 70/30 *w*/*w* (DES1/w) are listed in Table 3. It is observed that the variation of the three independent variables (X_1_, X_2_, X_3_) affected the dependent (Y_1_, Y_2_, Y_3_) ones. The TCC (Y_1_) fluctuated between 1.868 and 3.709 mg g^–1^_DW_, and the TPC (Y_2_) between 7.468 and 12.768 mg_GAE_ g^–1^_DW._ The IC50 (Y_3_) exhibited a minimum value of 0.118 and a maximum of 0.332 g_DW_ mL^–1^_sol_. The greatest TCC value was exhibited by the extract of Run 16 (T = 60 °C, t = 13.5 h, r = 30:1 g_SW_g^−1^_DW_), while for the extract of Run 18 (T = 60 °C, t = 3 h, r = 40:1 g_SW_g^−1^_DW_), the highest TPC and the lowest IC50 values were found. On the contrary, for the extract of Run 4 (T = 30 °C, t = 24 h, r = 40:1 g_SW_g^−1^_DW_), the highest IC50 was measured, rendering it the least potent one among the eighteen, as far as the antioxidant activity is concerned. The lowest carotenoids content was reported for the *C. vulgaris* extract of Run 3 (T = 30 °C, t = 3 h, r = 20:1 g_SW_g^−1^_DW_), while that of Run 13 (T = 45 °C, t = 3 h, r = 30:1 g_SW_g^−1^_DW_) showed the lowest phenolic content.

### 2.4. Statistical Analysis of Experimental Design Results

In order to draw conclusions about the responses’ dependence on the factors, a regression analysis of the experimental design results was carried out. Moreover, the ANOVA test was used to evaluate the resulting regression models. The statistical analysis of experimental data led to the development of reduced quadratic multiple regression models for each of the three responses investigated. 

The second-order polynomial quadratic functions of the TCC (Y_1_), TPC (Y_2_), and IC50 (Y_3_) and the factors T (X_1_), t (X_2_), and r (X_3_) are shown below:

(1) $$Y_{1}=-1.84295+0.081819\cdot X_{1}+0.166893\cdot X_{2}+0.078320\cdot X_{3}-0.001606\cdot X_{1}\cdot X_{2}-0.001170\cdot X_{1}\cdot X_{3}-0.003314\cdot X_{2}^{2}$$

(2) $$Y_{2}=25.65774-0.150048\cdot X_{1}+0.206034\cdot X_{2}-1.18459\cdot X_{3}+0.007376\cdot X_{1}\cdot X_{3}-0.006498\cdot X_{2}^{2}+0.015426\cdot X_{3}^{2}$$

(3) $$Y_{3}=0.0467107+0.000450\cdot X_{1}-0.004219\cdot X_{2}-0.013056\cdot X_{3}-0.000128\cdot X_{1}\cdot X_{3}+0.000190\cdot X_{2}\cdot X_{3}+0.000271\cdot X_{3}^{2}$$

Details regarding the ANOVA results are included in Table 4. ANOVA investigation indicated that all models were significant and accurate since their F-values were high and their p-values were lower than 0.0001 (Table 4). A factor is considered impactful to a given response when *p* < 0.005, hence it is concluded that TCC and TPC were most influenced by temperature (X_1_) and solvent-to-biomass ratio (X_3_), while the antioxidant activity was most affected by temperature (X_1_). Moreover, for TCC, two interaction terms (X_1_X_2_ and X_1_X_3_) and one quadratic $X_{2}^{2}$ were also significant, and for TPC one interaction term (X_1_X_3_) and two quadratics $X_{2}^{2}$, $X_{3}^{2}$. Non-significant lack of fit was found for all the developed predictive models. 

The models’ precision accuracy measures, which are also included in Table 4, indicated that the predictive models were reliable since their R^2^ values were greater than 0.9, and their R^2^ Adjusted, and R^2^ Predicted values were in reasonable agreement, i.e., the difference between them was less than 0.2. Moreover, values of adequate precision greater than 4 indicate that a model can be used to navigate the design space, something that was confirmed for all the obtained models of the present work. 

### 2.5. Study of the Factors’ Combined Effects 

**Figure 1:**
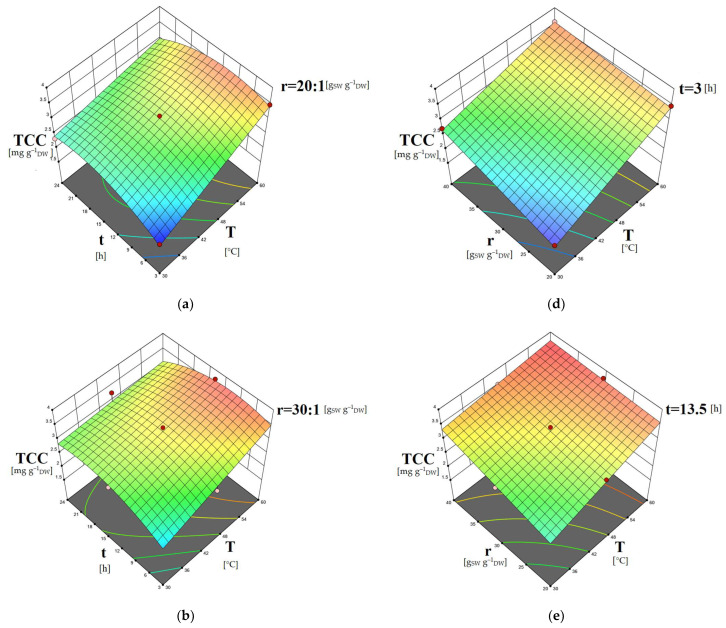

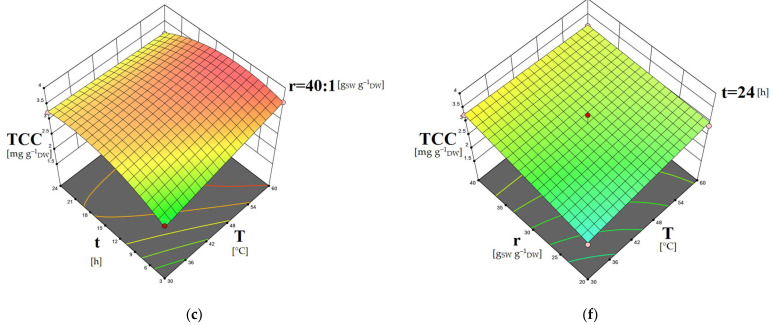
Three-dimensional surface plots showing the combined effects of temperature and time (**a**–**c**) and temperature and solvent-to-biomass ratio (**d**–**f**) on TCC [mg g^–1^_DW_ ] of the *C. vulgaris* extracts obtained using DES1/w solvent.

**Figure 2:**
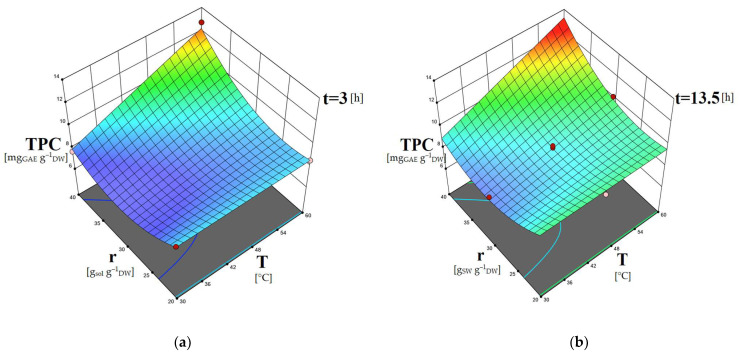

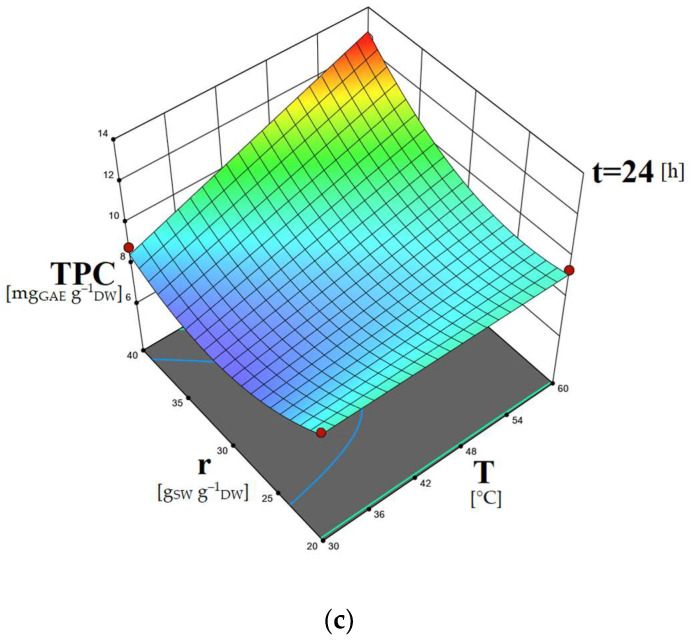
Three-dimensional surface plots showing the combined effects of temperature and solvent–biomass ratio (**a**–**c**) on TPC [mg_GAE_g^–1^_DW_] of the *C. vulgaris* extracts obtained using DES1/w solvent.

**Figure 3:**
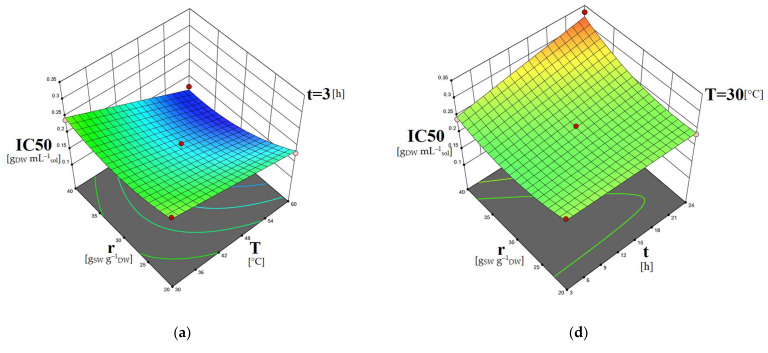

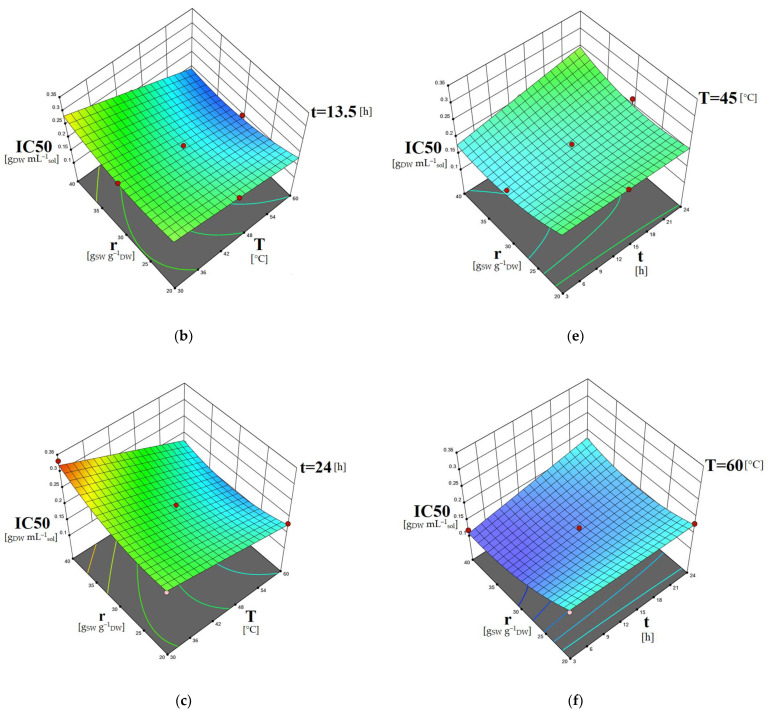
Three-dimensional surface plots showing the combined effects of (**a**–**c**) temperature and solvent-to-biomass ratio and (**d**–**f**) time and solvent-to-biomass ratio on the IC50 [g_DW_ mL^–1^_sol_] of the *C. vulgaris* extracts obtained using DES1/w solvent.

The 3D surface plots obtained by the models can contribute to the investigation of the interactions between the different independent variables regarding their effect on the dependent ones. In Figure 1, the combined effects of temperature and time, as well as temperature and solvent-to-biomass ratio on the carotenoid content, are presented. In Figure 2, the dependence of the phenolic content on the extraction time and solvent-to-biomass ratio is shown. The dependence of the extracts’ IC50 values on extraction temperature combined with a solvent-to-biomass ratio, as well as extraction time combined with solvent-to-biomass ratio, is depicted in Figure 3.

The extraction should last long enough to achieve effective contact of the solvent with the biomass, saturation of the biomass, as well as diffusion of the target biomolecules from the biomass to the solvent. Consequently, when performing solid–liquid extractions, the application of limited extraction times cannot assist remarkably in the recovery of biomolecules from their natural sources [54,55]. Figure 1a–c and Figure 2a–c indicate that the application of low extraction time (3 h) facilitated the least recovery of carotenoids and phenolics. Consequently, increasing the extraction duration is expected to give rise to the extraction of the solutes of interest [56]. By Figure 1a–c, it is evident that increasing extraction time assisted the carotenoids’ extraction; however, from a certain point, it did not contribute to a further increase in the TCC values. Particularly, it is observed in Figure 1d–f that for all T-r combinations, a rise from t = 3 h to t = 13.5 h augmented the TCC values, while a further increase to t = 24 h resulted in their reduction. Similar are the results for the phenolics recovery as shown in Figure 2a–c. These findings imply that the extracted carotenoids and phenolics sustained degradation under prolonged exposure to oxygen and light [49,55]. An initially increasing trend of the carotenoids content, followed by a decreasing one versus extraction time, was also reported for the *C. vulgaris* extractions using a conventional solvent [37]. A maximum extraction time above which the TPC of the *C. vulgaris* extracts decreases due to oxidation of the phenolics compounds was found by Mahmood et al., 2019 [40]; and Zakaria et al., 2017 [55], as well.

Higher extraction temperature facilitates the mass transport phenomena of the biomolecules of interest from the biomass cells to the solvent, as well as their solubilization [54,57]. By Figure 1d–f, it is observed that rising T and r positively impacted the carotenoids recovery. It can be supported that the concentration gradient of the specific biomolecules between the *C. vulgaris* cells and the solvent is increased with increasing solvent-to-biomass ratio [40]. This phenomenon, combined with the helpful influence of increasing temperature contributed to the acceleration of the carotenoids’ diffusion from the cells to the liquid phase and enabled their solubilization, resulting in greater TCC values for these extracts. Regarding the phenolics recovery (Figure 2a–c), according to the aforementioned mechanism of the combined effect of T-r, the beneficial impact of the rising extraction temperature is pronounced only under higher solvent-to-biomass ratio values.

Figure 3a–c indicate that the extracts obtained at low extraction temperature (30 °C) combined with a high solvent-to-biomass ratio (40:1 g_SW_ g^−1^_DW_) had the worst antioxidant activity. According to the analysis that preceded, at low extraction temperatures, extracts with a relatively lower content of carotenoids are obtained. Moreover, the application of a high solvent-to-biomass ratio, i.e., the use of a larger amount of solvent at a low extraction temperature, might have led to the extraction of other molecules present in the *C. vulgaris* cells that did not contribute to the antioxidant activity of the extracts [37]. Due to the presence of these compounds, along with the lower carotenoid concentrations, the extracts’ antioxidant potency was reduced, and higher IC50 values were measured. Furthermore, Figure 3d–f show that increasing extraction temperature enhances the antioxidant capacity of the obtained extracts for all r-t combinations. This finding could be attributed to the increased carotenoids and phenolics contents obtained under higher extraction temperatures. Figure 3a–c show also that the increase in extraction time for all r-T combinations negatively impacted the extracts’ antioxidant activity. As explained, prolonged extraction durations had a negative effect on the recovered carotenoids and phenolics integrity due to oxidative reactions favored by long exposure to air and light. 

### 2.6. Experimental Validation of the Models

The predictive capacity of the models was validated experimentally. Specifically, two different extractions of *C. vulgaris* with DES1/w were carried out under conditions that were chosen randomly to avoid any bias (Table 5). The experimental procedure and the analysis of the obtained extracts were performed in the exact same way that was employed for the implementation of the experimental design. The experimental TCC, TPC, and IC50 values of the extracts and their predicted values, which were calculated by using the aforementioned models (Equations (1)–(3)), are presented in Table 5. 

It is observed that the calculated TCC, TPC, and IC50 values agree very well with the experimentally measured ones. Hence, it is concluded that the models reproduce the experimental results satisfactorily, and thus, they can be safely used for prediction purposes within the range of the examined conditions.

### 2.7. Optimization of Extraction Process 

**Figure 4:**
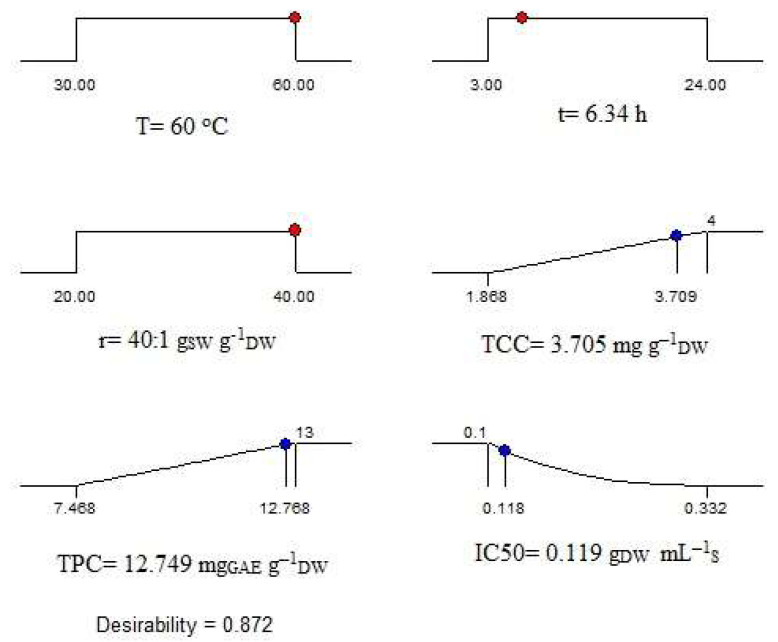
Optimization of the *C. vulgaris* biomass extraction using the solvent DES1/w.

The optimum extraction conditions of *C. vulgaris* biomass using DES1/w were defined by employing Design–Expert Ver. 13.0.5.0 software using the models of Equations (1)–(3). The independent variables, i.e., extraction temperature (X_1_), time (X_2_), and solvent-to-biomass ratio (X_3_), were set to vary within the ranges that were initially chosen for each one of them for the implementation of the experimental design. The responses TCC (Y_1_) and TPC (Y_2_) were set to maximize, while IC50 (Y_3_) was to minimize. Moreover, a weight factor of 1 was applied for TCC and TPC and 2.5 for IC50. The weight factors resulted from the optimization study, according to which the extracts’ antioxidant activity had to obtain a greater weight factor in comparison to the other two responses in order to obtain an overall optimized solution. The described optimization process and its results are depicted graphically in Figure 4. 

As seen, the predicted optimum conditions for the simultaneous maximization of TCC and TPC and minimization of IC50 were T = 60 °C, t = 6.34 h, and r = 40:1 g_SW_ g^−1^_DW_, under which the dependent variables TCC, TPC, and IC50 were determined 3.705 mg g^–1^_DW_, 12.749 mg_GAE_ g^–1^_DW_ and 0.119 g_DW_ mL^–1^_S_, respectively. Consequently, the highest temperature (60 °C) and solvent-to-biomass ratio (40:1 g_SW_ g^−1^_DW_), and an extraction time in-between the lowest (3 h) and intermediate (13.5 h) values that were studied were concluded to be the most convenient extraction conditions for the purpose of the present work. By comparing the values of the three studied responses obtained under the optimum conditions to the experimental ones in Table 3, it is seen that the recovered carotenoids yield is a bit lower than the experimental maximum. This small compromise was inevitable in order to obtain an overall optimized process. 

## 3. Materials and Methods

### 3.1. Chemicals 

The chemical reagents used for the preparation of the DESs and the extracts’ analyses were all of the analytical grade. Details regarding their provider and purity degree are included in Table 6. 

### 3.2. Microalgae Culture

The microalga *Chlorella Vulgaris* UTEX 1809 was obtained from the Algal Culture Collection at the University of Texas, Austin, USA. Autotrophic cultivation of *C. vulgaris* was performed in a 12 L stirred tank photobioreactor with a 10 L working volume (Bioengineering, Switzerland) continuously illuminated with cool white light at 65 μmol m^−2^sec^−1^ using light emitting diodes (LED). The temperature was maintained at 25 °C, and the stirring speed at 150 rpm. The bioreactor was filled with 9 L of Bold’s modified basal medium (BBM) containing NaNO_3_ (250 mg L^−1^), KH_2_PO_4_ (175 mg L^−1^), K_2_HPO_4_ (75 mg L^−1^), NaCl (25 mg L^−1^), MgSO_4_ 7H_2_O (75 mg L^−1^), anhydrous EDTA (50 mg L^−1^), CaCl_2_ 2H_2_O (25 mg L^−1^), FeSO_4_ 7H_2_O (4.98 mg L^−1^), MnCl_2_ 4H_2_O (1.44 mgL^−1^), ZnSO_4_ 7H_2_O (8.82 mg L^−1^), CuSO_4_ 5H_2_O (1.57 mg L^−1^), and KOH (31 mg L^−1^). It was then autoclaved at 120 °C for 20 min. Then, 1 L preculture of microalgal cells grown in 250 mL Erlenmeyer flasks under the same conditions on an orbital shaker at 180 rpm was used as inoculum. The pH at the beginning of the culture was adjusted to 6.8, and the sterile air supply to 0.25 vvm. After 15 days of cultivation, the microalgal biomass reached over 1.5 g L^−1^ dry weight. The liquid algal culture was concentrated by centrifugation (5000 rpm, for 10 min), and the concentrated algal mass was stored at 4 °C until used in further experiments.

### 3.3. Preparation of DESs 

The synthesis of the DESs was conducted by mixing their two components under a determined molar ratio using constant magnetic agitation at 80 °C until a clear and homogeneous liquid was formed (3–4 h). Three different DES systems were obtained by using choline chloride (ChCl) as HBA and the polyols 1,2 butanediol, glycerol, and ethylene glycol as HBD, at molar ratios 1:4, 1:2, and 1:2, respectively (Table 7). The DES constituents were selected due to their biodegradability, biocompatibility, availability, and relatively low cost. After synthesis, the DESs were dried in an oven at 50 °C under vacuum for 24 h. Subsequently, they were stored in dark in a desiccator. 

### 3.4. Measurement of DES Physical Properties 

Dynamic viscosity and density measurements for all pure DES and their mixtures with water (70/30 *w*/*w*) were conducted at 60 °C. A Brookfield digital viscometer (LV–DVI–E) connected to a thermostatic bath was employed for the viscosity measurements. An SC4–13R chamber was used to carry the solvent sample, along with an SC4–18 spindle attached to the moving shaft of the viscometer. Density measurements were performed with a KEM KYOTO density/specific gravity meter (DA–640). Prior to measurements, all DESs were dried in an oven at 50 °C under vacuum for 1 h to eliminate any adsorbed water. All measurements were performed in triplicate.

### 3.5. Extraction Process

Mixtures of ChCl:1,2 butanediol (1:4), ChCl:ethylene glycol (1:2), and ChCl:glycerol (1:2) with water (70/30 *w*/*w*) were employed as extraction solvents. According to the literature [16,58,59,60], the addition of water at this concentration reduces the viscosity enough without negatively affecting the hydrogen bond interactions between the constituents of the DES. S. Rozas et al., 2021 [60], in particular, concluded that in the case of ChCl:glycerol (1:2), a 10 to 30 wt% water content does not affect the main properties of the DES. The ethanol/water 70/30 *w*/*w* mixture (EtOH/w), a non-toxic, efficient, conventional solvent, was used for comparison [49]. The extraction conditions for the solvent screening were selected based on the relevant published work of our scientific group [37,49]. Specifically, the extractions were carried out at a 20:1 solvent-to-biomass ratio (r, [g_SW_ g^–1^_DW_]) for a time period of three hours (t, [h]). Biomass and solvents were weighed and added in a stoppled, double-walled glass vial that was connected to a thermostatic bath to keep the extraction temperature at 60 °C. Constant magnetic agitation was employed for the stirring of the biomass–solvent mixture. Upon the completion of the extractions, the resulting mixture was centrifuged at 4430 rcf for 10 min to separate the extract from the biomass. Subsequently, the extracts’ total carotenoid (TCC, [mg g^–1^_DW_]) and phenolic (TPC, [mg_GAE_ g^–1^_DW_]) contents, as well as their antioxidant activity (IC50, [g_DW_ mL^–1^_sol_]) were determined. 

Whereupon the solvent screening process, the most convenient one for the purpose of our study, was employed for further investigation using the same experimental configuration and procedure. Particularly, three of the most influential extraction parameters, temperature (T = 30–60 °C), solvent-to-biomass ratio (r = 20:1–40:1 g_sol_ g^–1^_DW_), and time (t = 3–24 h) were studied regarding their effect on the extracts’ total carotenoids content, total phenolics content and on their antioxidant capacity.

### 3.6. Determination of the Extracts’ Total Phenolic and Carotenoid Contents

Total phenolic content was estimated using the Folin–Ciocalteu reagent as described by Singleton et al., 1965 [61]. In particular, 7.9 mL of distilled water and 0.1 mL of extract were homogenized before the addition of 0.5 mL of Folin-Ciocalteu reagent. After vortexing, the resulting mixture, 1.5 mL Na_2_CO_3_ solution (20% *w*/*v*), was added, and the final mixture was incubated for 30 min in a water bath at 40 °C. Its absorbance was subsequently measured at 765 nm and compared to a gallic acid calibration curve. 

Total carotenoid content was determined according to the Association of Official Analytical Collaboration (AOAC) [62] methods. Following the extraction, the absorbance of 3 mL of extract was measured at 450 nm, and total carotenoids content was calculated from Equation (4) which was acquired by the β–carotene calibration curve:

TCC = 6.9691·Abs_450 nm_ − 0.1286 (4)

### 3.7. Determination of the Extracts’ Antioxidant Activity

The antioxidant activity of the extracts was assessed using the 2,2–Diphenyl–1–Picrylhydrazyl (DPPH) assay. In total, 100 μL of the extract was added to 3 mL of a DPPH ethanolic solution (0.03% *w*/*v*). The absorbance of the mixture was measured at 515 nm after its incubation for 20 min at room temperature. The calculated IC50 values refer to the sample concentration that is required to scavenge 50% of DPPH free radicals [63].

The spectrophotometric measurements were conducted In a SHIMADZU UV–1900, UV–VIS spectrophotometer. All measurements described above were conducted in triplicate, and the reported values of total carotenoids, total phenolics, and IC50 are the calculated average values.

### 3.8. Experimental Design and Statistical Analysis

Design–Expert Ver. 13.0.5.0 (Statease Inc., Minneapolis, MN, USA, test version) was employed for performing Response Surface Methodology (RSM) in order to optimize the extraction process of *C. vulgaris* biomass using DES-based solvents. A three-factor, three-level Central Composite Design (CCD) was followed. The influence of extraction temperature (X_1_), extraction time (X_2_), and solvent-to-biomass ratio (X_3_) on TCC (Y_1_), TPC (Y_2_), and the IC50 (Y_3_) value of the extracts was examined. Eighteen experiments in total, composed of six axial, eight factorial, and four central points, were realized randomly (Table 8). The obtained experimental data were subjected to regression analysis and Analysis of Variance (ANOVA). Thus, the determination of the significance of the influence of the independent variables (Factors) on every dependent variable (Response) was allowed, and fitting mathematical models were developed.

## 4. Conclusions

In this study, the extraction of *C. vulgaris* using DES-based solvents was examined. Among the three DESs that were synthesized in the present work, ChCl: glycerol (1:2) (DES2) was found to be the most viscus one, followed by ChCl:1,2 butanediol (1:4) (DES1) and ChCl: Ethylene glycol (1:2) (DES3). In order to overcome the drawback of high viscosity, which is essential for industrial applications, DES/water 70/30 *w*/*w* mixtures were also tested. Their viscosities followed the order that the pure DESs exhibited as well, i.e., DES2/w > DES1/w > DES3/w, but they were significantly lower than those of pure DESs. Regarding the measured densities of the pure DESs, their values diminish following the same order as the viscosity values. DES2/w and DES3/w demonstrated lower densities in comparison to pure DES2 and DES3, respectively, though this reduction with water addition was not significant. The density of DES1/w was found to be marginally higher than DES1.

From the solvent screening procedure, it was found that DES1/w solvent outperformed the two others, i.e., DES2/w and DES3/w, in extracting both carotenoid and phenolic compounds from *C. vulgaris* biomass. The TCC value measured at the DES1/w extract was 93.7% and 91.5% greater than the corresponding values measured for the DES2/w and DES3/w extracts, respectively. The TPC value that was determined for the DES1/w extract was almost double the TPC values found for the DES2/w and DES3/w extracts. It should be noted that all the DES-based solvents used in the present study performed significantly better in extracting phenolic compounds than carotenoids. DES1/w exhibited a higher phenolics extraction capacity than ethanol/water (control solvent) by approximately 10%. DES-based solvents follow the order DES1/w> DES2/w > DES3/w as per their antioxidant activity. According to the solvent screening results described above, it was concluded that the most convenient for the purpose of our study was DES1/w, and it was exploited for the *C. vulgaris* extraction process optimization.

To this purpose, a three-factor, three-level Central Composite Design (CCD) was performed, and the influence of the extraction parameters temperature (X_1_), time (X_2_), and solvent-to-biomass ratio (X_3_) on the responses TCC (Y_1_), TPC (Y_2_) and IC50 (Y_3_) was examined by performing 18 extractions. RSM and ANOVA assessment of the experimental data led to the development of reduced quadratic multiple regression models for each of the three dependent parameters that were studied. Non-significant lack of fit was found for all the developed models, and their predictive capacity was confirmed experimentally via two different experiments within the range of the examined experimental conditions. TCC and TPC were most influenced by temperature and solvent-to-biomass ratio, while the antioxidant activity was most affected by temperature. Carotenoid and phenolic extraction were enhanced under higher T and r values and increasing extraction temperature boosted the extracts’ antioxidant capacity. The increase in extraction time had a negative impact on the extracts’ antioxidant activity due to the oxidation of the recovered carotenoids and phenolics under long exposure to air and light. Τhe highest TCC and TPC and the lowest IC50 values were found at the temperature of 60 °C, a solvent-to-biomass mass ratio of 40:1, and extraction time of about six and a half hours.

## Figures and Tables

**Table 1:** Viscosities and densities of DESs and their 70/30 *w*/*w* water mixtures at 60 °C.

Solvent	Viscosity, η [cP]	Density, ϱ [g cm^−3^]
DES1	15.01 ± 0.04	1.0120 ± 0.0001
DES2	53.07 ± 0.22	1.1725 ± 0.0001
DES3	13.49 ± 0.11	1.0965 ± 0.0001
DES1/w	3.54 ± 0.01	1.0145 ± 0.0001
DES2/w	4.34 ± 0.02	1.1179 ± 0.0001
DES3/w	2.78 ± 0.05	1.0678 ± 0.0001

**Table 2:** TCC, TPC, and IC50 values measured in *C. vulgaris* extracts obtained at T = 60 °C, r = 20:1 g_SW_ g^−1^_DW,_ and t = 3 h using the DES-based solvents and EtOH/w. The acronym SW stands for solvent weight, and DW for dry weight biomass.

Solvent	TCC [mg g^–1^_DW_]	TPC [mg_GAE_ g^–1^_DW_]	IC50 [g_DW_ mL^–1^_sol_]
DES1/w	3.462 ± 0.121	8.553 ± 0.213	0.180 ± 0.011
DES2/w	0.218 ± 0.011	4.407 ± 0.128	0.260 ± 0.014
DES3/w	0.293 ± 0.014	4.687 ± 0.131	0.360 ± 0.018
EtOH/w	8.436 ± 0.211	7.686 ± 0.219	0.139 ± 0.010

**Table 3:** Experimental results of *C. vulgaris* extraction with DES1/w regarding TCC, TPC, and IC50.

Run	Experimental Design Conditions			Experimental Results		
	X_1_: T [°C]	X_2_: t [h]	X_3_: r [g_SW_ g^−1^_DW_]	Y_1_: TCC [mg g^–1^_DW_]	Y_2_: TPC [mg_GAE_ g^–1^_DW_]	Y_3_: IC50 [g_DW_ mL^–1^_sol_]
1	45	13.5	20:1	3.102	9.257	0.215
2	45	13.5	40:1	3.517	10.787	0.207
3	30	3	20:1	1.868	8.696	0.255
4	30	24	40:1	3.257	8.897	0.332
5	60	24	20:1	2.872	9.438	0.181
6	45	13.5	30:1	3.268	8.904	0.201
7	30	24	20:1	2.332	9.383	0.237
8	30	3	40:1	2.721	7.667	0.241
9	45	24	30:1	3.256	8.058	0.220
10	60	3	20:1	3.462	8.553	0.180
11	45	13.5	30:1	3.152	8.643	0.166
12	45	13.5	30:1	3.415	9.074	0.164
13	45	3	30:1	2.693	7.468	0.189
14	60	24	40:1	3.137	12.586	0.170
15	30	13.5	30:1	2.818	8.062	0.241
16	60	13.5	30:1	3.709	10.131	0.147
17	45	13.5	30:1	3.277	8.894	0.164
18	60	3	40:1	3.571	12.768	0.118

**Table 4:** Analysis of variance (ANOVA) and measures of the model’s prediction accuracy.

RESPONSE Y_1_-TCC					
Source	Sum of Squares	df	Mean Square	F-Value	*p*-Value
					Prob > F
**Model**	3.45	6	0.5750	31.55	<0.0001
$X_{1}$-**T**	1.41	1	1.41	77.36	<0.0001
$X_{2}$-**t**	0.0291	1	0.0291	1.59	0.2329
$X_{3}$-**r**	0.6589	1	0.6589	36.15	<0.0001
$X_{1}$ $X_{2}$	0.5121	1	0.5121	28.10	0.0003
$X_{1}$ $X_{3}$	0.2464	1	0.2464	13.52	0.0036
$X_{2}^{2}$	0.5932	1	0.5932	32.55	0.0001
**Residual**	0.2005	11	0.0182		
**Lack of fit**	0.1657	8	0.0207	1.79	0.3432
**Std. Dev.**	0.1350		**R^2^**	0.9451	
**Mean**	3.08		**Adj R^2^**	0.9151	
**C.V. %**	4.38		**Pred R^2^**	0.8464	
			**Adeq Precision**	22.0389	
**RESPONSE Y_2_-TPC**					
**Source**	**Sum of Squares**	**df**	**Mean Square**	**F-Value**	***p*-Value**
					**Prob > F**
**Model**	35.39	6	5.90	47.85	<0.0001
$X_{1}$-**T**	11.61	1	11.61	94.16	<0.0001
$X_{2}$-**t**	1.03	1	1.03	8.37	0.0146
$X_{3}$-**r**	5.45	1	5.45	44.19	<0.0001
$X_{1}$ $X_{3}$	9.85	1	9.85	79.87	<0.0001
$X_{2}^{2}$	1.59	1	1.59	12.90	0.0042
$X_{3}^{2}$	7.38	1	7.38	59.85	<0.0001
**Residual**	1.36	11	0.1233		
**Lack of fit**	1.26	8	0.1577	5.00	0.1064
**Std. Dev.**	0.3511		**R^2^**	0.9631	
**Mean**	9.29		**Adj R^2^**	0.9430	
**C.V. %**	3.78		**Pred R^2^**	0.8731	
			**Adeq Precision**	23.5207	
**RESPONSE Y_3_-IC50**					
**Source**	**Sum of Squares**	**df**	**Mean Square**	**F-Value**	***p*-Value**
					**Prob > F**
**Model**	0.0379	6	0.0063	23.39	<0.0001
$X_{1}$-**T**	0.0260	1	0.0260	96.30	<0.0001
$X_{2}$-**t**	0.0025	1	0.0025	9.13	0.0116
$X_{3}$-**r**	0.0000	1	0.0000	0.0000	1.0000
$X_{1}$ $X_{3}$	0.0030	1	0.0030	10.98	0.0069
$X_{2}$ $X_{3}$	0.0032	1	0.0032	11.85	0.0055
$X_{3}^{2}$	0.0033	1	0.0033	12.08	0.0052
**Residual**	0.0030	11	0.0003		
**Lack of fit**	0.0020	8	0.0002	0.7473	0.6714
**Std. Dev.**	0.0164		**R^2^**	0.9273	
**Mean**	0.2016		**Adj R^2^**	0.8877	
**C.V. %**	8.15		**Pred R^2^**	0.8156	
			**Adeq Precision**	20.676	

**Table 5:** Experimental conditions of the extractions conducted for models’ validation and the corresponding experimental and calculated TCC, TPC, and IC50 values.

Extraction	Experimental Values			Calculated Values		
	TCC [mg g^–1^_DW_]	TPC [mg_GAE_ g^–1^_DW_]	IC50 [g_DW_ mL^–1^_sol_]	TCC [mg g^–1^_DW_]	TPC [mg_GAE_ g^–1^_DW_]	IC50 [g_DW_ mL^–1^_sol_]
**1** (30 °C, 6 h, 20:1 g_SW_ g^−1^_DW_)	1.927	9.103	0.238	2.069	9.075	0.249
**2** (45 °C, 24 h, 20:1 g_SW_ g^−1^_DW_)	2.706	8.256	0.194	2.714	9.243	0.209

**Table 6:** Chemical reagents.

Chemical Reagents	Provider	Purity
2,2–Diphenyl–1–picrylhydrazyl	Alfa Aesar	95%
Folin Ciocalteu’s reagent	Carlo Erba reagents	Special grade
Methanol	Fisher Scientific	≥99.8%
Ethanol	Fisher Scientific	≥99.8%
Water	Fisher Scientific	HPLC grade
Choline chloride	Sigma Aldrich	≥98%
1,2 Butanediol	Sigma Aldrich	98%
Glycerol	Sigma Aldrich	≥99.0%
Ethylene glycol	Sigma Aldrich	99.8%
*β*–carotene	Alfa Aesar	99%
Gallic acid	Acros Organics	98%

**Table 7:** Synthesized DESs and their abbreviation.

DES	HBA	HBD	HBA:HBD Ratio
DES1	choline chloride	1,2 butanediol	1:4
DES2		glycerol	1:2
DES3		ethylene glycol	1:2

**Table 8:** Experimental design of three-factor, three-level CCD.

Run	Space Type	Factor 1/Level	Factor 2/Level	Factor 3/Level
		X_1_: T [°C]	X_2_: t [h]	X_3_: r [g_sol_ g^–1^_DW_]
1	Axial	45/0	13.5/0	20/–1
2	Axial	45/0	13.5/0	40/+1
3	Factorial	30/–1	3/–1	20/–1
4	Factorial	30/–1	24/+1	40/+1
5	Factorial	60/+1	24/+1	20/–1
6	Center	45/0	13.5/0	30/0
7	Factorial	30/–1	24/+1	20/–1
8	Factorial	30/–1	3/–1	40/+1
9	Axial	45/0	24/+1	30/0
10	Factorial	60/+1	3/–1	20/–1
11	Center	45/0	13.5/0	30/0
12	Center	45/0	13.5/0	30/0
13	Axial	45/0	3/–1	30/0
14	Factorial	60/+1	24/+1	40/+1
15	Axial	30/–1	13.5/0	30/0
16	Axial	60/+1	13.5/0	30/0
17	Center	45/0	13.5/0	30/0
18	Factorial	60/+1	3/–1	40/+1

## Data Availability

Not applicable.

## Sample Availability

Samples of the compounds are available from the authors.
